# Hypertension and the Fat-Soluble Vitamins A, D and E

**DOI:** 10.3390/ijerph120302793

**Published:** 2015-03-04

**Authors:** Agustin Llopis-González, Nuria Rubio-López, Monica Pineda-Alonso, Juan Carlos Martín-Escudero, Felipe Javier Chaves, Maximino Redondo, Maria Morales-Suarez-Varela

**Affiliations:** 1 Unit of Public Health, Hygiene and Environmental Health, Department of Preventive Medicine and Public Health, Food Science, Toxicology and Legal Medicine, University of Valencia, 46100 Valencia, Spain; E-Mails: nrubiolopez@hotmail.com (N.R.-L.); maria.m.morales@uv.es (M.M.-S.-V.); 2 CIBER Epidemiología y Salud Pública (CIBERESP), 28029 Madrid, Spain; 3 Center for Advanced Research in Public Health (CSISP-FISABIO), 46010 Valencia, Spain; 4 Internal Medicine Department, Rio Hortega University Hospital, 47012 Valladolid, Spain; E-Mails: monicpi16@gmail.com (M.P.A.); juancarlos.martinescudero@gmail.com (J.C.M.-E.); 5 Genotyping and Genetic Diagnosis Unit; Hospital Clinic Research Foundation and INCLIVA, University of Valencia, 46010 Valencia, Spain; E-Mail: felipe.chaves@uv.es; 6 CIBER Diabetes y Enferemedades Metabolicas Asociadas (CIBERDEM), 28029 Madrid, Spain; 7 Biochemistry Department, Agencia Sanitaria Costa del Sol, University of Málaga, Red de Investigación en Servicios de Salud en Enfermedades Crónicas (REDISSEC), 29603 Marbella, Málaga, Spain; E-Mail: mredondo@hcs.es

**Keywords:** hypertension, fat-soluble vitamin, nutritional deficiency

## Abstract

Hypertension affects populations globally and is thus a public health and socio-economic problem. Macronutrient and micronutrient deficiencies are common in the general population, and may be even more prevalent in hypertensive patients. This study aimed to determine a possible association between hypertension and intake of fat-soluble vitamins A, D and E. Participants were from the cross-sectional Hortega nutrition study conducted with a random sample of 1514 people (50.3% women, 49.7% men) and two groups: nonhypertensive controls ≥40 years old (n = 429; 28.3%); unknown untreated hypertension cases ≥40 years old (n = 246; 16.2%). Biochemical and anthropometric measurements were taken. Data on dietary intakes, education, socio-economic status, place of residence, health habits, comorbidities, alcohol consumption and smoking were collected and assessed. A descriptive data study was done and compared by ANOVA and Chi-Square. No *p* value higher than 0.05 was considered significant. The results showed that vitamin A intake was higher in the hypertensive subpopulation (1732.77 ± 962.27 µg *vs.* 1655.89 ± 902.81 µg), and vitamin D and E intakes were lower (8.13 ± 9.71 µg *vs.* 8.25 ± 9.52 µg and 18.79 ± 7.84 mg *vs.* 18.60 ± 8.20 mg, respectively). No statistically significant differences were found in any adjusted model. This study did not significantly associate intake of vitamins A, D and E with hypertension in people aged over 40. Future studies on this topic and a larger sample are necessary.

## 1. Introduction

Arterial hypertension (AHT) has become a major public health problem in developed countries. Together with hypercholesterolemia and smoking, it represents a major cardiovascular risk factor, whose high prevalence and ability can be modified by therapeutic intervention and lifestyle changes. All these factors render it an extremely interesting public health and socio-economic issue [1], especially since some studies have indicated that by 2025, prevalence of AHT will affect 29% of all adults worldwide [2]. It has been assumed that good hypertension control could prevent many cardiovascular diseases, while lack of such control may consequently lead to a higher incidence of hospitalization and mortality from associated diseases, which together would mean higher health and social services costs.

Hypertension is the result of a genetics-environmental interaction. Macronutrients and micronutrients are crucial for regulating blood pressure. The optimal combination of macronutrients and micronutrients has a significant impact on preventing and treating hypertension, in combination with drug therapy [3]. Early hypertension identification can help lower morbidity and mortality through cardiovascular disease (CD). The optimal age for hypertension screening is as of 40 years [4].

As the most cost-effective form, primary prevention can be accomplished mainly by reducing obesity, increasing physical activity, reducing alcohol intake to under 20–30 g per day, and eating a healthy low-salt diet, and sufficient potassium, fruits, vegetables, low-fat dairy products and minimum unsaturated fats [5]. A possible positive effect of antioxidant vitamins (E, C) has also been investigated, as reflected by different studies [6,7]. Failure in primary prevention results in society spending larger amounts on health. Even a modest reduction in AHT can have good public health benefits as it can help not only cut its incidence, but also control AHT subjects, and prevent major cardiovascular events and other associated diseases [8,9].

Different works [10] reflect a drop in blood pressure (BP) levels with a vegetarian diet: here as responsible agents for a drop in BP we find a high fiber content, low-fat content or high mineral content, such as potassium and magnesium, and low salt, as well as a possible effect of antioxidant vitamins (E, C).

Vitamins are a group of organic compounds that differ from one another in terms of their chemical composition, which the organism requires in small amounts to carry out specific metabolic functions within cells. Basically, we distinguish hydrosoluble vitamins and fat-soluble vitamins, such as vitamins A, D and E.

Vitamin A and its analogs are important regulators of cell proliferation, differentiation and cell apoptosis, and are involved in immune functions. Retinoic acid is involved in embryonic kidney development by controlling branching morphogenesis [11,12]. Nonphysiological levels of vitamin A in pregnant women affect the organogenesis of newborns by lowering the number of nephrons in kidneys, which predisposes to future hypertension development in adulthood [5,11,13,14]. No studies have investigated the influence of vitamin A on hypertension development. Vitamin D deficiency can result from lack of exposure to ultraviolet B (UVB) radiation from the sun, which can lead to its limited production by the skin. One hypothesis states that if low levels of vitamin D can cause high BP, then supplementation of this vitamin might lower it as antihypertensive effects of vitamin D occur in patients with high BP and vitamin D deficiency; thus vitamin D supplementation may improve AHT [15,16,17,18]. Vitamin D hypovitaminosis is an independent risk factor of mortality in the general population. A meta-analysis published in 2013 showed that long-term vitamin D supplementation is associated with a significant reduction in mortality [19,20]. Vitamin D could also influence vascular biology by modulating endothelial function and inflammatory status [21]. All of this spells a significant issue because older adults are more susceptible to both vitamin D insufficiency and increased risk of CD [22]. Vitamin E (α-tocopherol) is a fat-soluble vitamin and one of the most important natural antioxidants [6] with several cardio-protective effects, such as reducing oxidative stress, increasing glucose utilization by insulin and improving the endothelial function of vessels [23]. A combination of antioxidant vitamins containing vitamin E, vitamin C, β-carotene, and zinc taken by hypertensive patients for 8 weeks has been described to lower BP and to increase nitric oxide metabolites in urine [24,25].

Finally as numerous studies have shown, nutrition plays a key role in health; specifically vitamins are essential and necessary micronutrients that are involved in numerous vital functions. In the literature, we have found independent studies on vitamins A,D and E, but none conducted together. These vitamins present anti-inflammatory and antioxidant effects, and their low serum concentration may be an important factor in early stages of the pathogenesis of many chronic diseases, such as hypertension [26,27]. We hypothesized that low levels of vitamins A, D and E are related to hypertension in adults. The objective of this study was to determine a possible association between hypertension and fat-soluble vitamins A, D and E intake in a cross-sectional study conducted in the city of Valladolid (Spain).

## 2. Materials and Methods

### 2.1. Study Design

We grounded our investigation on the results of the cross-sectional “Hortega Study” [28,29]; a two-stage, nested, population-based, epidemiological case-control monitoring pilot study carried out in an adult population sample in the province of Valladolid (Spain). We randomly selected 20% (n = 35,901) of the population and stratified it according to hypertension and non-hypertension, and also to age and gender. People aged 90 years or more, nonresidents in the province, patients with clear cognitive impairment and terminal patients were excluded. This screening left a sample of 34,742 people. Using uniform age- and sex-stratified random sampling, a representative subsample of the general population was selected (n = 1514). We used a broad battery of nutritional and health-related parameters.

### 2.2. Study Population

**Figure 1:**
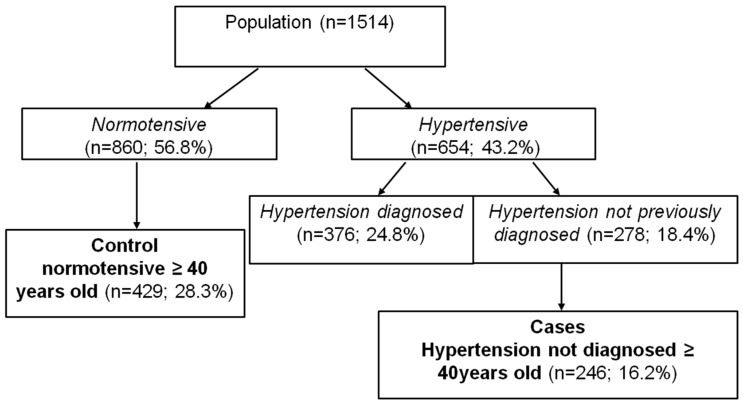
Flow diagram of the selection of study subpopulations.

We started with a sample of 1514 people in a population aged 21–89 years; 50.3% were women and 49.7% were men. BP was taken and, following the WHO criteria [30], the sample was divided into AHT (n = 654; 43.2%) and non-AHT (n = 860; 56.8%). In the AHT subpopulation, those patients not previously diagnosed with AHT were selected (n = 278; 18.4%). Finally, both subpopulations were selected from individuals aged over 40, which left a sample of 429 non-AHT individuals (controls) (28.3%) and 246 (16.2%) AHT patients (cases) (Figure 1).

### 2.3. Patient Data

To carry out the study, information about age and gender was collected. Patients underwent a physical examination during which anthropometric variables BMI and waist/hip ratio and BP were measured (as recommended by the Joint National Committee [31] with an automatic HEM-711DLX IntelliSense, Automatic Blood Pressure Monitor, OMRON HEALTHCARE, China, validated and calibrated for this study). A blood sample was taken to determine different biochemical parameters (fasting glucose, triglycerides, HDL-cholesterol, LDL-cholesterol, total cholesterol) and existence of comorbidities was assessed. Data on medications used (anti-hypertensive, anti-diabetics and anti-hypolipidemics) were also collected.

A nutritional survey consisting of three 24-hours recalls done on three different days and a semi-quantitative Food Frequency Questionnaire were conducted, while photographs of portions were collected to assess the intake of various dietary components. The average intake levels of various dietary components were also assessed by taking the average of the three 24-hours recalls and the semi-quantitative Food Frequency Questionnaire. The 24-hour recalls were transferred to the ‘Alimentación y Salud’ (Diet and Health) program developed by the Institute of Nutrition and Food Technology, University of Granada [32] (Spain), which specified each food item eaten. This program then estimated the respective nutrients. We finally obtained the total energy and micronutrients intake specifically for vitamins A, D and E.

### 2.4. Epidemiological Analysis

Categorical variables were expressed as percentages and continuous variables were reported as means (SD) for the normally distributed parameters, or as median and interquartile ranges. For the data analysis, a descriptive data study was performed with the IBM SPSS software (SPSS, version 17.0; SPSS Inc., Chicago, IL, USA). The mean values, and absolute and relative frequencies of the different variables were obtained in the two subpopulations and within each one after stratifying for gender. The data from the two subpopulations (total and gender) were compared by ANOVA (quantitative) and Chi-Square (qualitative). No *p* value higher than 0.05 was considered significant. In a second step, the analytical data study was performed by a logistic regression analysis to estimate a possible association between presence of AHT and intake of vitamins A, D or E.

The variables of the intake of these fat-soluble vitamins were categorized according to whether they met the recommended daily intake (RDI) or were lower or higher than RDI. Individuals’ age and gender were taken into account (Table 1) and estimated using a logistic regression bivariate value and an unadjusted odds ratio (ORc) for all the intake categories of each vitamin compared to the reference category.

**Table 1:** Recommended Daily Intake values of vitamins A, D and E for a Spanish population according to age and gender.

Gender	Age (years)	Vitamin A (µg)	Vitamin D (µg)	Vitamin E (µg)
Women	40–49	800	5	8
	50–59	800	5	8
	60–69	800	10	8
	≥70	700	15	10
Men	40–49	1000	5	10
	50–59	1000	5	10
	60–69	1000	10	10
	≥70	900	15	12

Notes: Taken from: Ortega *et al*.: Recommended daily intakes of energy and nutrients for the Spanish population. Madrid: Department of Nutrition. Universidad Complutense de Madrid; 2004 [33].

A multivariate logistic regression analysis was done by designing a set of adjustment models with the different variables included, with which the adjusted odds ratio (ORa1, ORa2 and ORa3) were calculated for each fat-soluble vitamin and category (lower RDI or higher RDI).

In a first step, age, gender and body mass index (BMI) were included with variables ‘age + gender + BMI’. ORa1: gender + age (every 10 years) + BMI (continuous). In a next step, comorbidities, such as presence or absence of diabetes mellitus (DM) and hypercholesterolemia, were included in the adjusted model. ORa2: gender + age (every 10 years) + BMI (continuous) + DM (yes/no) + hypercholesterolemia (yes/no). In a third step, the model was adjusted to fit the variables: smoking, alcohol consumption, exercise performed and energy intake. ORa3: gender + age (every 10 years) + BMI (continuous) + DM (yes/no) + hypercholesterolemia (yes/no) + smoking (smoker/ex-smoker/nonsmoker) + alcohol intake (0, >0 to 5, >5 to 15, >15 g of alcohol/day), exercise (METS total continuous) daily energy intake (kcal, continuous).

## 3. Results

### 3.1. General Characteristics and Anthropometrical Variables

As shown in Table 2, the total hypertension prevalence of the study population aged over 40 years in this study was 43.2%, of whom 18.4% were newly diagnosed cases. In the 40–59 age group, non-hypertensive individuals (non-AHT: women 67.42%, men 61.45%; AHT: women 23.89%, men 35.34%) predominated in both women and men. Moreover in those aged over 70 years, the percentage of AHT in men and women was much higher compared to non-AHT, with statistically significant differences in both cases (non-AHT: women 22.62%, men 28.37%; AHT: women 59.29%, men 54.89%). However, this was not observed in the 60–69 year-old age group.

Regarding weight and BMI, statistically significant higher values were obtained for women with AHT, and BMI values were 28.66 ± 4.99 and 25.36 ± 3.58, respectively. No statistically significant differences were observed for men between the values of these AHT and non-AHT variables. The only criteria followed were that the male population yielded a *p*-value under 0.05 for the waist/hip ratio when this value in men with AHT was higher than in non-AHT individuals. When attaching importance to these findings, both the BMI and waist/hip ratios become major factors, which can lead to obesity, a factor closely connected to hypertension.

Among all the non-AHT individuals, one man and one woman on diuretic-type anti-hypertension treatment (prescribed for kidney pathology), and among the AHT subjects, only 4.4% of women and 3.8% of men in both cases, were 80% IECAS and 20% diuretics. We also identified a greater requirement of antidiabetic and hypolipidemic medications among AHT subjects. However we found no statistically significant differences with non-AHT, nor for gender.

**Table 2:** General characteristics and anthropometric variables.

Variable		Non-AHT (n = 429; 63.6%)				AHT (n = 246; 36.4%)			
		Women (n = 221; 51.5%)		Men (n = 208; 48.5%)		Women (n = 113; 45.9%)		Men (n = 133; 54.1%)	
		N	%/Mean ± SD	N	%/Mean ± SD	N	%/Mean ± SD	N	%/Mean ± SD
Age	40–59	149	67.42 *	128	61.54 *	27	23.89 *	47	35.34 *
	60–69	22	9.95	21	10.10	19	16.81	13	9.77
	>70	50	22.62 *	59	28.37 *	67	59.29 *	73	54.89*
Weight (kg)			62.21 ± 8.84 *	201	76.44 ± 10.78	111	66.82 ± 12.96 *	129	77.09 ± 12.01
BMI			25.36 ± 3.58 *	208	26.75 ± 3.22	112	28.66 ± 4.99 *	133	27.38 ± 3.57
Waist/hip			0.81 ± 0.09	206	0.94 ± 0.07 *	110	0.84 ± 0.10	130	0.96 ± 0.06 *
Medication									
Anti-HTA									
No medication			99.5	207	99.5	108	95.6	128	96.2
On medication			0.5	1	0.5	5	4.4	5	3.8
IECAs			0	0	0	4	80	4	80
Diuretics			100	1	100	1	20	1	20
Anti-DM									
No medication			99.1	199	95.7	103	91.2	126	94.7
On medication			0.9	9	0.3	10	8.8	7	5.3
Oral antidiabetics			50	4	44.4	4	40	3	42.9
Insulin			50	5	55.6	6	60	4	57.1
Anti-hypolipidemics									
No medication			97.3	196	94.2	104	92.0	123	92.5
On medication			2.7	12	5.8	9	8.0	10	7.5

Notes: * *p*-value ≤ 0.05 (Chi-square; ANOVA test); AHT: Arterial hypertension; SD: Standard Deviation; BMI: Body Mass Index.

### 3.2. Blood Pressure, Biochemical Parameters and Vitamins Intake

In Table 3, we observe the BP and biochemical parameters: the mean SBP and DBP were generally lower in non-AHT (women SBP: 118.64 ± 12.11 mmHg; DBP: 75.70 ± 7.66 mmHg and men SBP: 123.51 ± 10.44 mmHg; DBP: 76.41 ± 6.86 mmHg) than in AHT (women SBP: 152.00 ± 15.42 mmHg; DBP: 87.30 ± 9.40 mmHg and men SBP: 150.61 ± 15.90 mmHg; DBP: 87.13 ± 10.05 mmHg). In all cases, statistically significant differences were obtained.

For the mean triglycerides values, significant differences for both women and men were observed, with higher mean values found in the AHT subpopulation (women: 165.64 ± 85.30 mg/dL; men: 228.25 ± 152.83 mg/dL) compared to the non-AHT subpopulations (women 142.04 ± 67.31 mg/dL; men: 212.02 ± 107.89 mg/dL). We observed no statistically differences between AHT and non-AHT in relation to total energy intake.

For the mean values of fasting glucose, that is, in men with AHT (98.62 ± 24.94 mg/dL) and women with AHT (94.03 ± 14.40 mg/dL), we observed statistically significant differences compared to their corresponding subpopulations of non-AHT individuals (men 92.30 ± 18.33 mg/dL; women 88.77 ± 12.17 mg/dL). Prevalences of DM, kidney damage, metabolic syndrome and hyper-triglyceridemia were higher in the AHT patient group (for men and women) (*p* < 0.05).

**Table 3:** Blood pressure, biochemical parameters, vitamins intake and prevalence of comorbidities.

Variable	Non-AHT (n = 429; 63.6%)				AHT (n = 246; 36.4%)			
	Women (n = 221; 51.5%)		Men (n = 208; 48.5%)		Women (n = 113; 45.9%)		Men (n = 133; 54.1%)	
	N	%/Mean ± SD	N	%/Mean ± SD	N	%/Mean ± SD	N	%/Mean ± SD
Systolic blood pressure (mmHg)	218	118.64 ± 12.11 *	207	123.51 ± 10.44 *	112	152.00 ± 15.42 *	132	150.61 ± 15.90 *
Diastolic blood pressure (mmHg)	218	75.70 ± 7.66 *	207	76.41 ± 6.86 *	112	87.30 ± 9.40 *	132	87.13 ± 10.05 *
Triglycerides (mg/dL)	221	142.04 ± 67.31 *	208	212.02 ± 107.89	113	165.64 ± 85.30 *	133	228.25 ± 152.83
HDL cholesterol (mg/dL)	221	59.11 ± 13.58	208	45.79 ± 11.88	113	56.25 ± 14.48	133	47.64 ± 12.24
LDL cholesterol (mg/dL)	221	122.94 ± 32.58	208	121.79 ± 33.06	113	128.98 ± 33.79	133	117.68 ± 35.03
Total cholesterol (mg/dL)	221	210.47 ± 37.35	208	209.98 ± 34.66	113	218.36 ± 36.01	133	210.97 ± 41.73
Fasting glucose (mg/dL)	221	88.77 ± 12.17 *	208	92.30 ± 18.3 3*	113	94.03 ± 14.40 *	133	98.62 ± 24.94 *
Diabetes mellitus	3	1.4 *	11	5.3 *	10	8.8 *	15	11.3 *
Kidney damage	5	2.3 *	4	1.9 *	10	9.0 *	13	9.8 *
Metabolic syndrome	25	11.3 *	41	19.7 *	40	35.4 *	53	39.8 *
Hypertriglyceridemia	108	50.5	108	55.1	51	49.0	58	47.2
Hypercholesterolemia	29	13.2 *	38	18.3	27	23.9 *	22	16.5
Obesity	35	17.0 *	49	24.3	46	41.4 *	35	26.7
Overweight	102	49.5 *	151	74.8	85	76.6 *	99	75.6
Total energy (kcal/day)	221	4311.22 ± 1546.59	208	4566.67 ± 1623.22	113	4650.59 ± 1470.62	133	4564.06 ± 1680.60
VITAMINS: Vit. A (µg/day)	221	1650.03 ± 908.85 *	208	1661.76 ± 899.54	113	1977.00 ± 1016.82 *	133	1533.96 ± 870.43
Vit. D (µg/day)	221	7.70 ± 9.76	208	8.68 ± 9.29	113	7.96 ± 9.93	133	8.27 ± 9.57
Vit. E (µg/day)	221	17.91 ± 8.22	208	19.27 ± 8.30	113	18.44 ± 7.48	133	19.12 ± 8.19

Notes: * *p*-value ≤ 0.05 (Chi-square; ANOVA test); AHT: Arterial hypertension; HDL: High Density Lipoproteins; LDL: Low Density Lipoproteins.

**Table 4:** Comorbidities and vitamin intake.

Variable	Non-AHT (n = 429; 63.6%)				AHT (n = 246; 36.4%)			
		Vitamin A	Vitamin D	Vitamin E		Vitamin A	Vitamin D	Vitamin E
	N	Mean (µg) ± SD	Mean (µg) ± SD	Mean (mg) ± SD	N	Mean (µg) ± SD	Mean (µg) ± SD	Mean (mg) ± SD
Diabetes mellitus	14	2688.55 ± 2065.74 *	16.61 ± 50.11	19.73 ± 9.25	35	1808.94 ± 820.34 *	32.64 ± 64.98	21.80 ± 7.47
Kidney damage	9	868.85 ± 797.70	3.45 ± 3.61	10.11 ± 7.04 *	23	2830.17 ± 5826.18	8.47 ± 12.12	17.56 ± 8.68 *
Metabolic syndrome	66	2617.44 ± 4422.13	12.39 ± 29.83	19.40 ± 10.15	93	2100.90 ± 3068.78	13.14 ± 44.83	18.72 ± 12.86
Hypertriglyceridemia	216	2327.85 ± 3792.64	11.20 ± 27.83	18.95 ± 10.61	109	2149.84 ± 2876.60	18.01 ± 52.52	19.62 ± 12.32
Hypercholesterolemia	67	2170.14 ± 2712.68	10.027 ± 14.22	18.58 ± 11.14	49	2712.68 ± 3997.04	15.67 ± 40.59	20.41 ± 12.22
Obesity	84	1609.50 ± 2051.38	7.09 ± 10.80 *	16.66 ± 8.34	81	2260.91 ± 3711.34	16.24 ± 40.14 *	18.17 ± 9.39
Overweight	253	2200.86 ± 3547.93	11.64 ± 26.03	19.33 ± 10.78	184	2003.96 ± 2575.37	13.74 ± 31.56	18.35 ± 9.91

Notes: * *p*-value ≤ 0.05 (Chi-square; ANOVA test); AHT: Arterial hypertension; SD: Standard Deviation.

No statistically significant differences were observed between the mean values of total cholesterol, high density lipoproteins (HDL) and low density lipoproteins (LDL) cholesterol between the AHT and non-AHT populations for both genders.

Regarding daily vitamin intake, differences were observed for vitamin A between the two female subpopulations, and the average value was higher in the AHT population (AHT 1977.00 ± 1016.82 mg; non-AHT 1650.03 ± 908.85 mg). In all the other cases, no p-values under 0.05 were obtained.

Based on the results in Table 4, we can state that the non-AHT patients diagnosed with DM reported a significantly higher intake of vitamin A compared to AHT (2688.55 ± 2065.74 µg *vs.* 1808.94 ± 820.34 µg). This suggests that higher vitamin A intake might have protective effects. In the group of patients with kidney damage, vitamin E intake levels were higher among AHT patients than among non-AHT ones. We found no differences in intake of vitamin A, D and E in the groups of patients with metabolic syndrome, hypertriglyceridemia and hypercholesterolemia, nor between AHT and non-AHT patients. However, patients with obesity and AHT presented high levels of vitamin D intake.

For kidney damage, intake of the studied vitamins was generally higher in the AHT subpopulation, with statistically significant differences for vitamin E (10.11 ± 7.04 mg *vs.* 17.56 ± 8.68 mg). Finally for obesity cases, vitamin E intake was significantly higher in the AHT subpopulation (16.24 ± 40.14 µg *vs.* 7.09 ± 0.80 µg). In all the other cases, no statistically significant differences were found.

### 3.3. Prevalence of Comorbidities

Table 3 also provides the results of prevalence of comorbidities; in the female population, prevalence was generally higher in the AHT subpopulation compared to non-AHT: DM (8.8% AHT *vs.* 1.4% non-AHT), kidney damage (9.0% AHT *vs.* 2.3% non-AHT), metabolic syndrome (35.4% AHT *vs.* 11.3% non-AHT), hypercholesterolemia (23.9% AHT *vs.* 13.2% non-AHT), obesity (41.4% AHT *vs.* 17.0% non-AHT) and overweight (76.6% AHT *vs.* 49.5% non-AHT), and with statistically significant differences.

In the male subpopulation, statistically significant differences were obtained when comparing prevalence of DM (11.3% AHT *vs.* 8.8% non-AHT), kidney damage (9.8% AHT *vs.* 1.9% non-AHT) and metabolic syndrome (39.8% AHT *vs.* 19.7% non-AHT), and these were higher in the subpopulation of hypertensive men.

No significant differences were found neither between sexes nor between the AHT and non-AHT subpopulations for hypertriglyceridemia (women 49.0% AHT *vs.* 50.5% non-AHT; men 47.2% AHT *vs.* 55.1% non-AHT) and hypercholesterolemia (women 23.9% AHT *vs.* 13.2% non-AHT; men 16.5% AHT *vs.* 18.3% non-AHT).

The results obtained for the obesity and overweight measurements were interesting, as confirmed by factors in hypertension development, where significant differences were obtained for women (obesity 41.4% AHT *vs.* 17.0% non-AHT; overweight 76.6% AHT *vs.* 49.5% non-AHT), but not for men (obesity 26.7% AHT *vs.* 24.3% non-AHT; overweight: 75.6% AHT *vs.* 74.8% non-AHT).

### 3.4. Multivariate Analysis

Table 5 shows the results of the logistic regression analysis. In no ORc cases were statistically significant differences found in the values obtained, except for the ‘lower RDI’ vitamin D value, with a value of 0.47 (95%CI: 0.24–0.93). This suggests that daily intake below the RDI is negatively associated with presence of hypertension.

**Table 5:** Analysis of the bivariate and multivariate logistic regressions.

Variable		*RDI*	*Lower RDI*	*Higher RDI*
Vitamin A	Non-AHT (n (%))	36 (60.0)	34 (63.0)	250 (60.8)
	AHT (n (%))	24 (40.0)	20 (37.0)	161 (39.2)
	*ORc (95%CI)*	1.00 (ref)	0.97 (0.57–1.68)	0.88 (0.41–1.88)
	*ORa1 (95%CI)*	1.00 (ref)	1.08 (0.59–1.99)	0.91 (0.40–2.07)
	*ORa2 (95%CI)*	1.00 (ref)	1.17 (0.63–2.15)	1.80 (0.86–3.74)
	*ORa3 (95%CI)*	1.00 (ref)	1.29 (0.65–2.54)	1.55 (0.73–3.31)
Vitamin D	Non-AHT (n (%))	23 (53.5)	180 (57.9)	118 (71.1)
	AHT (n (%))	20 (46.5)	131 (42.1)	48 (28.9)
	*ORc (95%CI)*	1.00 (ref)	0.47 (0.24–0.93) *	0.84 (0.44–1.59)
	*ORa1 (95%CI)*	1.00 (ref)	1.77 (0.83–3.80)	1.71 (0.83–3.52)
	*ORa2 (95%CI)*	1.00 (ref)	1.85 (0.85–4.01)	1.80 (0.86–3.74)
	*ORa3 (95%CI)*	1.00 (ref)	1.91 (0.86–4.24)	1.55 (0.73–3.31)
Vitamin E	Non-AHT (n (%))	19 (65.5)	17 (60.7)	240 (66.3)
	AHT (n (%))	10 (34.5)	11 (39.3)	122 (33.7)
	*ORc (95%CI)*	1.00 (ref)	0.97 (0.44–2.14)	1.23 (0.42–3.61)
	*ORa1 (95%CI)*	1.00 (ref)	0.90 (0.36–2.25)	0.70 (0.20–2.39)
	*ORa2 (95%CI)*	1.00 (ref)	0.91 (0.36–2.26)	0.73 (0.21–2.52)
	*ORa3 (95%CI)*	1.00 (ref)	1.04 (0.38–2.85)	0.78 (0.22–2.76)

Notes: * *p*-value ≤ 0.05 (Chi-square); RDI: Recommended Daily Intake; AHT: Arterial Hypertension; CI: Confidence Interval; ORa1: gender + age + BMI; ORa2: gender + age + BMI+ DM + hypercholesterolemia; ORa3: gender + age + BMI + DM + hypercholesterolemia + smoking + alcohol consumption + exercise + daily energy intake.

The ORa1 values obtained for vitamin A were similar to those of ORc. Regarding vitamin D, we noted that after including and adjusting variables, the trend obtained in the bivariate analysis reversed, with ORa1 above 1. However, we found no statistically significant CIs. Finally for vitamin E, we observed a change in the ‘higher RDI’ category, less than 1, but no statistically significant ORa1.

In all the ORa values, no statistically significant confidence intervals were obtained, which were stable for both vitamins D and E. For vitamin A, the ORa2 value was higher compared to the corresponding ORa1, especially in the ‘higher RDI’ category. P values were never under 0.05.

In all cases a trend was seen for the adjusted and crude models, except for the ‘lower RDI’ vitamin E category, where the ORa3 value was higher than 1, but the confidence interval included unit 1. Therefore based on these results, the possible association between the daily intake levels of these vitamins and presence of hypertension was not likely in our study population.

## 4. Discussion

In a community-based population with older subjects (aged over 40 years), we found a hypertension prevalence of 43.2%, of whom 16.2% had been recently diagnosed. For age, we observed more hypertensive individuals (men and women) in the group aged over 70 years, and AHT was associated with increased weight and BMI in women, and with a higher waist/hip ratio in men. Regarding the biochemical parameters, AHT was associated with an increased level of triglycerides and fasting glucose. In relation to comorbidities in AHT patients, although they were recently diagnosed cases, they were associated with increased DM, kidney damage and metabolic syndrome for both genders, and with hypercholesterolemia, obesity and overweight in women. We did not observe differences in vitamin D and E between AHT and non-AHT, but vitamin A was high in the AHT group (both men and women). This is possibly because the strategy to prevent hypertension is at the forefront to improve eating habits, which supports the low prescription frequency of anti-hypertensive treatments that we observed among hypertension patients. Despite there being newly diagnosed AHT cases, the increased presence of comorbidities in this group is particularly relevant.

For our epidemiological study, we opted for a case-control design which allowed us to assess or estimate the positive or negative association between various parameters, this being the objective of our study, and development of hypertension by calculating odds ratio (ORs). The selection criterion was BP, thus significant differences were found between groups for both the total sample and its distribution according to gender and age.

From the results obtained herein, we were unable to identify an association between fat-soluble vitamins A, D, E intake and AHT. However, we started with a discrepancy in the results observed in the literature review, clinical trials and studies done in experimental animals, which supports the involvement of vitamins E and C in lowering AHT or helping drug treatments thanks to their high antioxidant effect by acting synergistically on oxidative stress in AHT. Despite there being newly diagnosed cases of AHT, we were struck by the increased presence of comorbidities among them. We must also emphasize that we assessed vitamin intake, and one weakness of our study was that serum levels of these vitamins was lacking, so absorption may have been impaired.

As we hypothesized an association between AHT and intake of fat-soluble vitamins A, D and E, we decided to control the confounding factors that might have distorted the results. This is why we took into account anthropometric measurements, biochemical determinations, pathophysiological and other factors to help establish and assess the extent of this association.

The majority of the control subpopulation (non-AHT) fell into the 49–59 age group, while individuals aged over 70 years predominated in the hypertensive subpopulation. In this study we saw how the number of nondiagnosed cases of hypertension in the study population was 18.4%. This figure is high and must be taken into account given the importance of knowledge of hypertension and control in cardiovascular failure risk prevention. These results agree with those reported in the literature on BP rising with age (>60% over 60 years), particularly in hypertensive individuals.

The results of the anthropometric measurements revealed that the AHT subpopulation had higher BMI, weight and waist/hip ratios, of which the last meaasurement was more pronounced among AHT women. The positive association between body weight and BP has been also confirmed by the results obtained in the Intersalt study [34].

Continuous growth in obesity in the general population, along with a larger number of associated diseases such as DM, dyslipidemia or cardiovascular diseases, have established a direct relationship with increased AHT prevalence in society today. Therefore biochemical measurements were taken. From the results we should note that the mean values of triglycerides and fasting glucose were higher in the AHT subpopulation, and that AHT was a predictive variable of cardiovascular risk (according to the Spanish Society of Cardiology). We found no statistically significant differences between mean cholesterol values and our subpopulations, although both values were higher than recommended by the Spanish Society of Cardiology—200 mg/dL.

The comorbidity data collected from our population showed that AHT is associated with kidney damage, DM, metabolic syndrome, obesity and being overweight, with a higher prevalence in the AHT subpopulation and statistically significant differences. These findings coincide with conclusions drawn from other AHT prevalence studies conducted in different locations.

For macronutrients intake, whose values were obtained by averaging the data from the 24-hour recalls and the semi-quantitative Food Frequency Questionnaire, the results clearly show intake of proteins, carbohydrates and fats, which was higher in the AHT subpopulation than in the nonAHT subpopulation. Nonetheless the differences between them were not statistically significant, so we were unable to associate the higher intake of these nutrients with hypertension. Intake of vitamin A was significantly higher in non-AHT diabetic patients, as were vitamin E in AHT patients with kidney damage and vitamin D intake in obese AHT patients. Although these findings might suggest a correlation, further research needs to be conducted to confirm this association.

One of the limitations of this study was our assessment of nutrients and accuracy of the food intake data since no accurate method has yet been developed. In order to limit them in our study, we used records for three different days, and validated questionnaires and photographs to determine portion sizes. Furthermore, the averages of two different methods and validated software to transform food intake into nutrients and to calculate mean values were used. Another significant limitation of this study is that it lacks biomarker micronutrients (vitamins A,D and E) in serum.

The literature available on vitamin A and AHT has studied the importance of vitamin A on the genesis of AHT as this vitamin is involved in embryonic kidney development; therefore when deficient, the final number of nephrons may be manifested in adulthood as AHT [5,11,35,36]. However, we found no study that has been conducted on the influence of excess or deficient vitamin A intake in an adult AHT population. From the data obtained in our study population, we conclude that we cannot claim this association as we observed no statistically significant difference in vitamin intake between the two study subpopulations.

The results of studies and literature reviews on vitamin D mention an association between hypertension and vitamin D, and refer to a deficiency of this vitamin in individuals with AHT [17,18,37,38]. In these studies, we noted that the study population reported a deficient vitamin D intake, and that such supplementation could improve these individuals’ antihypertensive status. Yet other studies [39] have found no significant effect of vitamin D supplementation. No negative association has been observed between vitamin D and hypertension in individuals in whom the plasma concentrations of this vitamin gave normal values (40.00–80.00 ng/mL) [12]. Although we initially observed a significant ORc difference in our study for the ‘lower RDI’ category, which could mean a negative association between vitamin D and AHT, it was not relevant for ORa. Therefore, no significant association was found in either subset. According to the adjusted RDI mean values, vitamin D intake in both subpopulations was practically the same in both cases. So we assume that there was no association and it should, accordingly, not affect BP.

Studies on vitamin E and AHT [40] have been conducted to associate the antioxidant effect of this vitamin on oxidative stress in AHT. However, this effect is achieved with high levels of vitamin E (higher than 400 IU/day), which may prove toxic. Other studies have shown [41] that obesity in adolescents is associated with high levels of LDL and low plasma α-tocopherol content. Thus a combination of vitamin C and E has been used in most studies This combination exerts a hypotensive effect, even with a smaller amount of vitamin E, due to the synergy between them. In our population, we observed that our values fell within the recommended limits in both subpopulations, and we noted no statistically significant differences between subpopulations, which suggests no positive or negative association between vitamin E intake and AHT. If there was an association, it may possibly not have been observed because the intake levels of our population were not high enough to enable vitamin E to execute its antioxidant properties to lower BP.

Hypertension as a global problem needs to be effectively treated. The most effective way, and also by far the cheapest, is prevention to lower prevalence levels, and beginning by covering primary prevention from early ages, e.g., children and adolescents, is advised [42]. Investments in educating populations about healthy habits and proper nutrition, led by a Mediterranean diet, further promotion of the importance of daily physical activity, and reducing salt intake will be paid back by lowering the cost of treatment of developed hypertension and associated diseases.

## 5. Conclusions

This population-based study conducted in Spain indicates the importance of hypertension in populations aged over 40 years, specifically for intakes beyond the recommended levels of fat-soluble vitamins A and D, which has been identified to possibly increase the risk of hypertension, but not statistically significantly. However, this association has not been identified for vitamin E after adjusting for gender, age, BMI, diabetes mellitus, hypercholesterolemia, smoking, alcohol, exercise and daily energy intake. In this context, our results have not significantly associated the intake of vitamins A, D and E with hypertension in people aged over 40 years. Therefore, future studies on this topic and a larger sample are needed.
